# Gene Expression and Isoform Variation Analysis using Affymetrix Exon Arrays

**DOI:** 10.1186/1471-2164-9-529

**Published:** 2008-11-07

**Authors:** Amandine Bemmo, David Benovoy, Tony Kwan, Daniel J Gaffney, Roderick V Jensen, Jacek Majewski

**Affiliations:** 1 Universite de Montreal, Montreal, QC, Canada; 2 Department of Human Genetics, McGill University, Montreal, QC, Canada; 3 McGill University and Genome Quebec Innovation Center, Montreal, QC, Canada; 4 Department of Biological Sciences, Virginia Tech, Blacksburg, Virginia, USA

## Abstract

**Background:**

Alternative splicing and isoform level expression profiling is an emerging field of interest within genomics. Splicing sensitive microarrays, with probes targeted to individual exons or exon-junctions, are becoming increasingly popular as a tool capable of both expression profiling and finer scale isoform detection. Despite their intuitive appeal, relatively little is known about the performance of such tools, particularly in comparison with more traditional 3' targeted microarrays. Here, we use the well studied Microarray Quality Control (MAQC) dataset to benchmark the Affymetrix Exon Array, and compare it to two other popular platforms: Illumina, and Affymetrix U133.

**Results:**

We show that at the gene expression level, the Exon Array performs comparably with the two 3' targeted platforms. However, the interplatform correlation of the results is slightly lower than between the two 3' arrays. We show that some of the discrepancies stem from the RNA amplification protocols, e.g. the Exon Array is able to detect expression of non-polyadenylated transcripts. More importantly, we show that many other differences result from the ability of the Exon Array to monitor more detailed isoform-level changes; several examples illustrate that changes detected by the 3' platforms are actually isoform variations, and that the nature of these variations can be resolved using Exon Array data. Finally, we show how the Exon Array can be used to detect alternative isoform differences, such as alternative splicing, transcript termination, and alternative promoter usage. We discuss the possible pitfalls and false positives resulting from isoform-level analysis.

**Conclusion:**

The Exon Array is a valuable tool that can be used to profile gene expression while providing important additional information regarding the types of gene isoforms that are expressed and variable. However, analysis of alternative splicing requires much more hands on effort and visualization of results in order to correctly interpret the data, and generally results in considerably higher false positive rates than expression analysis. One of the main sources of error in the MAQC dataset is variation in amplification efficiency across transcripts, most likely caused by joint effects of elevated GC content in the 5' ends of genes and reduced likelihood of random-primed first strand synthesis in the 3' ends of genes. These effects are currently not adequately corrected using existing statistical methods. We outline approaches to reduce such errors by filtering out potentially problematic data.

## Background

Alternative pre-mRNA splicing is a process that allows for the production of numerous protein variants from a single genomic locus. As researchers are becoming aware of the importance of splicing and mRNA processing in generating transcriptomic diversity, isoform-sensitive microarrays are rapidly gaining popularity in gene expression analysis [1,2]. In particular, Affymetrix Exon Arrays are becoming a standard for both general and isoform-level expression analysis [3-11]. Briefly, the Exon Array platform relies on 25-mer oligonucleotide probes to target the individual exons of a gene. The expression level of each exon can be detected independently, and summarized to monitor transcript expression levels as well as changes of individual transcript isoforms. The more universal coverage of the "Whole-Transcript" (WT) arrays renders them an attractive alternative to the traditional 3' biased expression microarrays.

We have previously successfully used Exon Arrays to demonstrate variation in isoform level expression in human populations [12] and associate this variation with underlying genetic differences [8]. We showed that the Exon Array is indeed a powerful and flexible tool, allowing for the detection of changes in splicing, transcript initiation, and termination. However, analysis of exon-level data is considerably more complicated than traditional analysis of gene expression. The complexity of the analysis may prevent many researchers from using WT arrays and profiting from associated advances in gene expression profiling.

Here, we use the example of a well studied system to outline the analysis and present results of a typical Exon Array experiment. We use the brain and reference human mRNA samples previously studied by the MicroArray Quality Control (MAQC) consortium [13,14]. These commercially available samples provide a high quality reference dataset for comparing microarray results across various platforms and laboratories. The human brain has very distinct gene expression signatures, and the comparison with the reference (combined) tissue pool results in detection of numerous genes with differential expression levels. The original MAQC study relied on these samples to demonstrate high concordance between various microarray platforms. Incidentally, the human brain is also rich in specific isoforms, and constitutes a highly suitable system for assessing the performance of the Exon Array as both an expression and isoform-sensitive platform.

## Results

### Variability across labs

**Figure 1:**
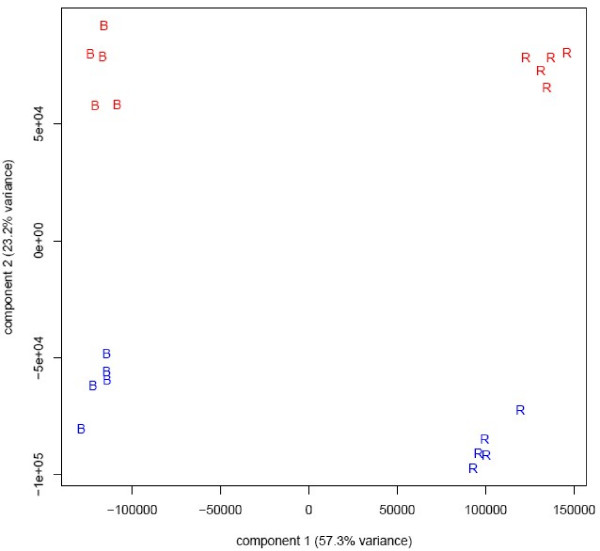
**PCA plots at the probe set level show two main sources of variation among the 20 samples**. The first principal component explains 65% of the variance and corresponds, as expected, to the biological source of the sample: brain (B) vs. reference (R). The second principal component explains 20% of the variance and corresponds to the "lab effect" between VT (blue), and McGill (red) – that is, it illustrates the technical variability across labs.

Five technical replicates of brain and reference were hybridized in two independent labs: McGill University (MU) and Virginia Tech (VT), for a total of 20 samples. Principal component analysis, which is a commonly used method to visualize sources of variability in the data, is shown in Figure 1. Our experience with Exon Arrays indicates that in general the ribosomal RNA reduction step is the most inconsistent part of the protocol and is likely to be a major contributor to the differences across labs.

**Figure 2:**
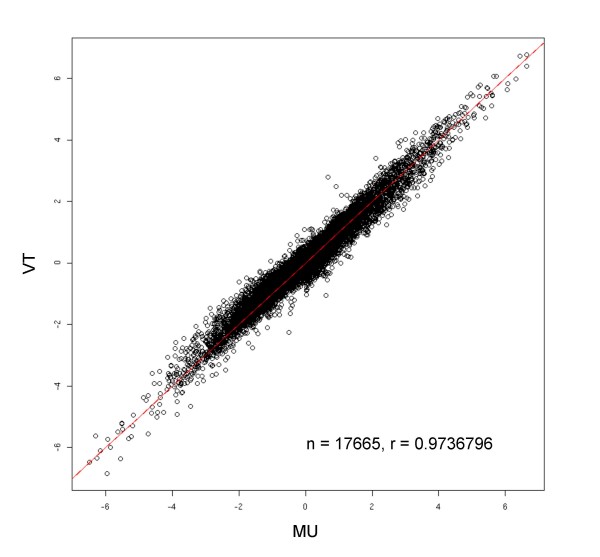
**Comparison of log_2_(FC) detected between the biological samples for the two labs**. Despite significant variation in expression measure across test sites, the fold change estimates are highly correlated.

Variability in hybridization intensities, background noise, and random errors across labs may contribute to differences in final conclusions resulting from microarray analyses. In the case of the MAQC data, the final goal was to quantify differences in gene expression levels between the human brain and reference tissues. A relevant metric of such expression difference is the fold change (FC), calculated as FC = Expression(Brain)/Expression(Reference). In Figure 2, we show a correlation plot comparing the calculated fold changes in genes expression between the two labs. Despite the inter-lab variability in expression levels shown in the PCA plots, the final results (fold changes) are highly consistent for the two labs, with a correlation coefficient of greater than 0.97.

### Variability across summarization methods

The aim of the summarization step in microarray analysis is generally to combine signals from multiple probes, which target the same expression unit, into a single expression index. Most of the popular methods strive for robustness against outlier probes (e.g. cross hybridizing, saturated, or non-responsive probes). We used our fold change results to compare two commonly used summarization methods: PLIER and RMA. We noted that RMA does result in a slight compression of fold changes, as has been observed in prior studies using other microarray platforms [13]. However, we find that the correlation of fold changes obtained from the two approaches is very high (r = 0.99).

### Variability across platforms

**Figure 3:**
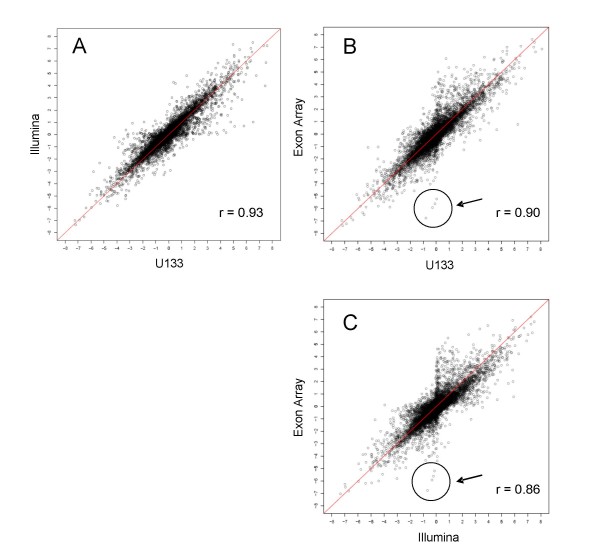
**Correlation of fold changes between Affymetrix U133, Illumina, and the Affymetrix Exon Array**. Fold changes (log_2 _transformed) between brain and reference expression levels for 8391 genes common to all three platforms: A) Illumina vs. U133. B) Exon Array vs. U133, C) Exon Array vs. Illumina. The arrow points to the highly discordant detection of 4 histone genes: *HIST1H3B, HIST1H1B, HIST1H3C, HIST1H3I*.

The original MAQC studies demonstrated that microarray results are highly consistent across different platforms [13]. In Figure 3, we compare the performance of the Exon Array in determining gene expression levels with two other popular platforms previously used by MAQC: Illumina Bead Array and Affymetrix U133 Gene Chip. In order to facilitate comparison across labs as well as platforms, we selected a number of genes which are reliably annotated and targeted by a common set of probesets (see Methods).

For the Exon Arrays, the fold changes were calculated by combining the results from the two labs (MU and VT). For the sake of consistency in the comparison, two test sites were chosen at random and combined for each platform within the MAQC dataset. We find that the 3' targeted platforms, Illumina Human-6 BeadChip and Affymetrix U133, produce the most consistent results (R = 0.92). This is not surprising, since the probe selection regions for the two platforms largely coincide, and the amplification protocols are poly-A primed and biased towards the 3' ends of genes. The correlation with the Exon Array is slightly lower: R = 0.89 for U133 and 0.85 for Illumina. It has been previously shown [15-17], that the Exon Arrays are effective tools for gene expression profiling. Therefore, it is of interest, to examine the main sources of differences between the Exon Arrays and other platforms. Thus, in the analysis below we will concentrate on the genes whose predicted expression patterns are not consistent across platforms. In particular, the Exon Array is able to distinguish between specific isoforms of a given genomic locus, whereas the Illumina and Affymetrix U133 platforms generally target only a single isoform.

### Alternative Isoform Detection

**Figure 4:**
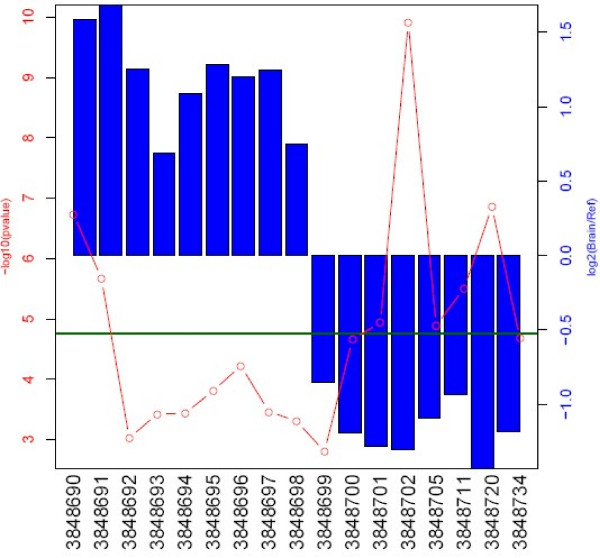
**Exon array analysis of the *ELAVL1 *gene expression differences between brain and reference tissues**. The horizontal scale corresponds to each probeset within the gene from the 3' to 5' ends. The height of the blue bars indicates the log_2_(fold change) in expression between the samples. The red line indicates statistical significance, -log_10_(p-value).

It has previously been pointed out that some discordant results in the original MAQC [13] study were caused by differential isoform expression, and differences in probe placement across platforms. One particular discordant gene, *ELAVL1*, was suspected to express two alternative isoforms, differing in the 3' UTR region. In Figure 4, we use the example of *ELAVL1 *to illustrate the advantages of using the Exon Array for profiling individual isoforms.

**Figure 5:**
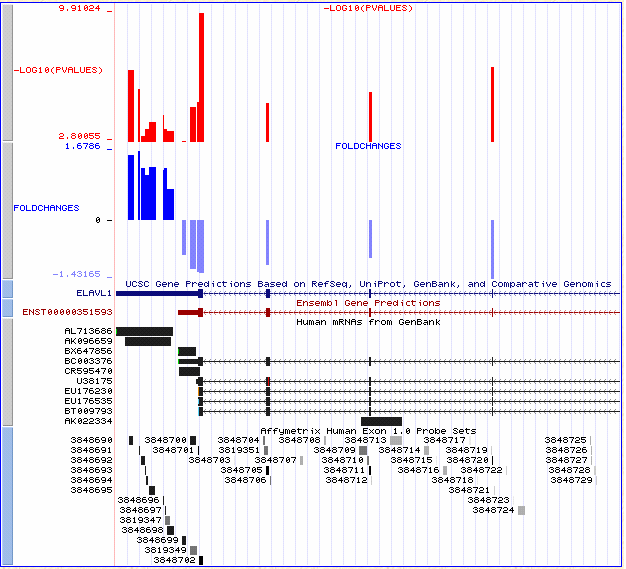
**Visualization of expression patterns of *ELAVL*1 gene**. The top two custom tracks display the Exon Array information from Figure 4: statistical significance and fold change. Note that the two probeset "blocks" correspond to the two isoforms of the gene. The long 3'UTR isoform is predominantly expressed in the brain, whereas the short isoform is more abundant in the reference tissues.

It is clear that although the Exon Array does not report the entire gene as differentially expressed, individual probesets within the gene reach high statistical significance levels (p < 10^-9^). More interestingly, the gene appears to be composed of two "blocks", with the first block on the 3' end showing elevated expression in the brain, while the second block has elevated expression in the reference sample. In order to understand the more precise nature of this isoform change, it is advantageous to visualize this data in the context of known gene annotation, EST, and mRNA data. Generally, our lab uses the custom track feature of the UCSC genome browser [18], in order to export our own information and combine it with publicly available data (Figure 5).

In Additional file 1, we present other examples of discordance between the platforms, further illustrating the value of additional information present on the Exon Array in profiling both "whole transcript" and "isoform-level" changes.

### Differences in Amplification and Labelling Protocols

The four most discordant genes between the 3' arrays and the WT array (see Figure 3) are histone genes: *HIST1H3B, HIST1H1B, HIST1H3C, HIST1H3I*, all of which are part of the histone gene cluster on chromosome 6p21.3. The Exon Array identifies those RNAs as over 50 fold less abundant in the brain than in the reference sample, while the 3' targeted platforms register no expression differences and very low overall expression levels. It has been shown that most histone genes lack a poly-A tail [19] and that the stability of such non-adenylated transcripts varies greatly with intracellular conditions such as those present in brain tissues [20]. Both Illumina and the Affymetrix U133 arrays use 3', poly-T primed RNA amplification protocols and do not detect histone gene expression. In contrast, the Exon Array uses WT random primed amplification, which does not necessitate the presence of a poly-A tail. The difference of histone RNA abundance is the most striking example of a result that is specifically detected by the Exon Array, but not the other platforms. However, there are many other such differences within the dataset (see Additional file 1).

### Using the Exon Array to Profile Alternative Isoforms

One of the biggest challenges in profiling alternative isoforms using Exon Arrays is the deconvolution of mRNA processing and transcription. A simple comparison of probeset intensities across samples is not sufficient; if an exon belongs to a transcript that is differentially expressed, the examination of a single exon out of its genomic context will lead to an incorrect conclusion. A very simple and intuitive solution to this problem is the use of the Splicing Index (SI), which is calculated by dividing the probe set intensity by the metaprobeset intensity (i.e. exon expression/gene expression), after the addition of a stabilization constant to both the probeset and meta-probeset scores [21]. This simple procedure normalizes the expression level of each exon and accounts for any possible gene expression differences between samples. However, we find that the splicing index has some undesirable statistical properties (arising from large errors in the estimates in both the numerator and the denominator) as well as being prone to methodological artefacts (see below), and should be used with caution. Thus, we have also used a simpler, but more labour intensive method, of carrying out the entire analysis at the probeset level, and relying on visualization and manual curation of the results in order to distinguish splicing and expression differences between samples. While more robust statistical approaches are being developed, we strongly advocate visualization of results in the context of genome annotation and EST evidence in order to filter out false positive signals. We have relied on custom scripts and modifications of the UCSC and ENSEMBL genome browsers, but increasingly useful and user-friendly commercial packages for the Exon Arrays are available (e.g. Partek Genomics Suite, Biotique XRay) along with academic BioConductor packages [22-24]. Below, we describe in more detail two approaches to alternative isoform detection. For the case of simplicity, only the core (most confident) subset of Exon Array probesets was considered in this analysis.

#### Probe set level analysis

At this level of the analysis, each probeset (roughly corresponding to an exon) is used as a unit of expression, instead of a meta probeset (a transcript) as is done in more traditional gene expression analysis. With appropriate statistical significance cut-offs (e.g. a Benjamini-Hochberg [25] False Discovery Rate correction), it is generally possible to select a highly confident set of probesets exhibiting significantly altered expression. However, it is not immediately possible to classify the "hits" as results of alternative isoform expression (e.g. alternative splicing), differential gene expression, or both. The easiest way of factoring out of gene expression is to consider only the genes whose expression does not change across samples or treatments. That is, we can select probesets that are statistically significant, but which belong to genes whose meta-probe set expression does not appear to be significantly altered (nominal p > 0.05). For the MAQC samples, we generated a list of the top 100 such genes. The list and links to the UCSC browser are provided in the Additional file 2. The top candidates show evidence for differential promoter usage, polyadenylation, and alternative splicing. A few examples appear to be annotation errors, where the Affymetrix annotation combines two distinct genes into a single transcript cluster. In general, we advocate RT-PCR based validation of alternative isoforms. However, cross validation with existing information is also extremely useful. Extensive EST and mRNA based information on tissue specific splicing is available from many sources, e.g. from the ASAPII [26] or Hollywood [27]. Most of the source data can be viewed directly in the UCSC genome browser by displaying the mRNA, spliced EST, or AltEvents tracks.

#### Splicing Index (SI) analysis

**Figure 6:**
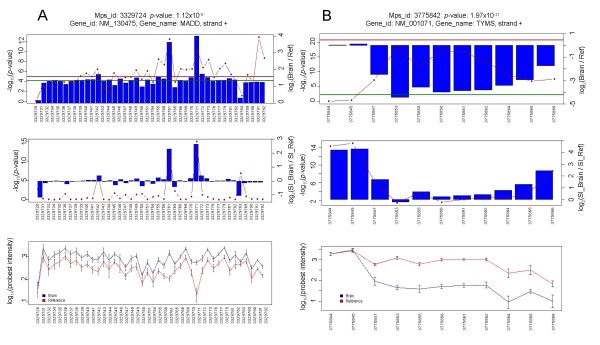
**Examples of Candidates from Splicing Index Analysis**. Top panels show the p-values (dotted line) and fold-changes (blue bars) for the expression of individual probesets. The centre panels show the values normalized for overall difference in gene expression (SI). Bottom panels show the raw hybridization levels of each probeset. A) MADD – successful use of the splicing index. In this example, in the presence of an overall 3-fold gene expression difference between the samples, the SI factors out the expression difference and indicates three alternatively spliced probesets – 3329761, 3329771, and 33291783 – all of which have strong supporting RefSeq annotation evidence for alternative splicing. B) TYMS – a typical false positive, where differences in probe response levels close to the edges of the transcript suggest alternative isoform usage. Such results are often erroneous, resulting from non-uniform response of individual probesets to large (in this case ~20 fold) changes in gene expression. Note the elevated signal intensity (bottom panel) at the 5' end of the gene, suggesting saturation, and a reduced intensity at the 3' terminus, possibly to reduced amplification efficiency.

SI is calculated by dividing the probe set intensity by the metaprobeset intensity. This simple procedure normalizes the expression level of each exon and should account for any possible gene expression differences between samples. An example of a successful use of SI analysis is illustrated in Figure 6A. Intuitively, the splicing index may be viewed as an approximate fractional inclusion level of a probeset within a transcript. However, we find many statistical and methodological problems arising from the use of the SI metric. Specifically, comparing SI values across samples makes the assumption that all probesets within a gene have comparable response (linear or log-linear) to changes in RNA levels. This assumption is generally violated, and hence SI comparisons result in high false positive rates. The most severe non-linearities in response are exhibited by probesets that are expressed close to the background levels, and probesets within highly expressed genes whose detection range is saturated. One of the most common methodological artefacts is illustrated in Figure 6B; probesets that are close to the 3' ends of genes are not amplified as efficiently as interior probesets while probesets close to the 5' end have elevated GC content and reduced specificity (see below). In addition, probesets that belong to skipped exons, which are included at low levels in both samples – i.e. these are actually alternatively spliced exons, but are NOT differentially spliced across samples. It should be noted that such artefacts are not limited to the use of the splicing index, and also applied to other commonly employed methods that attempt to correct for expression differences, such as the two-way ANOVA method implemented by Partek and Biotique XRay software. Some of the arising problems may be avoided by various filtering approaches; e.g. removing probesets with extremely high or low SI values, or probesets with extremely low coefficients of variation (possibly saturated). A more detailed discussion of these effects is presented at the Affymetrix website [21] and methods are being developed to enable these filtering criteria in an automated fashion [28]. Such approaches are likely to reduce false positive rates, at a cost of a reduced coverage of the genome. In Additional file 3 we present the top 100 candidates resulting from the SI analysis of the MAQC data, after filtering out all probesets expressed below background (average detection above background [DABG] p-value > 0.05).

### Edge Bias Effect

**Figure 7:**
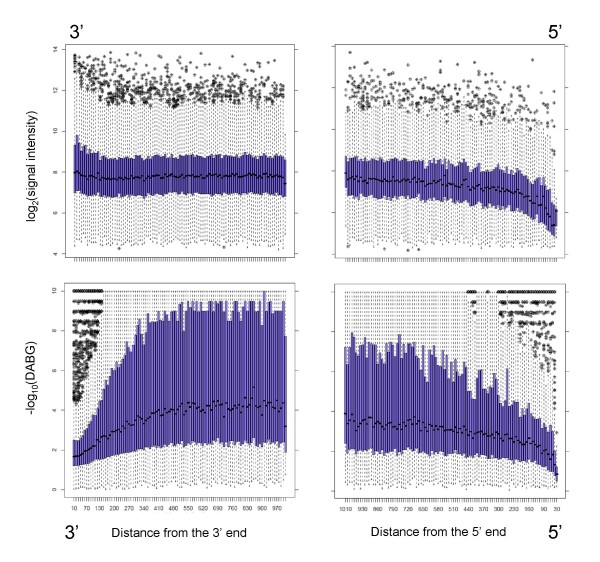
**Edge bias**. This figure illustrates variation of hybridization intensity across transcripts. For each probeset expressed above background levels, we determined the average hybridization intensity as a function of distance from the 5' and 3' ends of the mRNA molecule. Top panels show the average signal intensity as a function of probeset distance from the 5' and 3' ends of transcripts. A significant decrease in signal strength is seen at the 3' end, while a slight increase occurs at the 5' end. Bottom panels illustrate the ability of the array to detect the hybridization signal above background levels. Mean DABG values decrease at both 5' and 3' extremities of genes. The 3' effect results directly from the reduction in hybridization intensity. The 5' effect is most likely the result of increased GC content of the 5' probes located close to unmethylated gene promoters and CpG islands. Both effects cause false positive results in Splicing Index and Splicing ANOVA analyses in the presence of changes in expression of the whole transcript. Only genes with detectable expression (average DABG p-value < 0.05) and total mRNA length greater than 1000 nucleotides were included in this analysis. The values were calculated as log-averages of core probeset intensity across all samples. Each point on the plot corresponds to all probeset ending within a bin of length 10 bp, at the indicated distance from mRNA termini.

**Figure 8:**
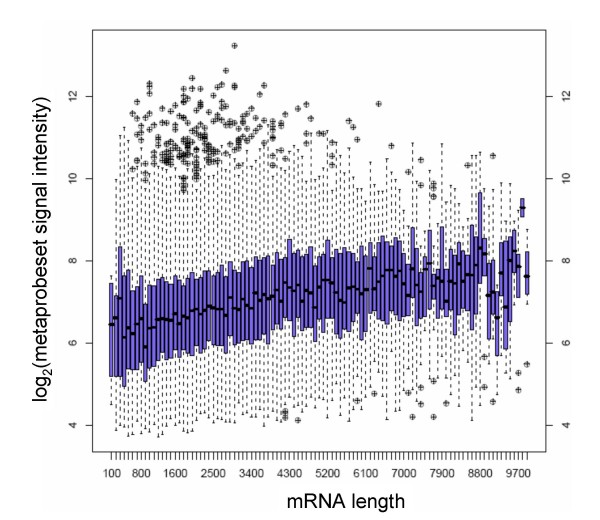
**Exon Array average gene expression index as a function of transcript (mRNA) length**. There is a highly significant positive correlation of expression and length (R = 0.18, p < 10^-20^). This effect is most likely an artefact of the edge bias illustrated in Figure 7; short transcripts have a lower overall efficiency of first strand synthesis and appear to be expressed at lower levels. The effect is not observed in the 3' amplified U133 (R = 0.05) and Illumina (R = -0.03) results.

In the course of the splicing index analysis described above, we noted an excess of "hits" occurring in the 3' and 5' regions of transcripts. We hypothesized that this effect could arise partly due to a bias during the first strand synthesis in the random primed amplification step used in exon array processing. Briefly, first strand synthesis proceeds from the 3' end to the 5' end of each transcript, initiating at random points along the mRNA molecule. Each probeset in the interior of the mRNA is likely to be represented by multiple randomly primed initiation events. However, probesets towards the 3' end of the mRNA have a lower chance of coverage – simply because the molecule ends and priming cannot occur at any point downstream of the 3' end. In order to test this hypothesis and quantify the possible biases, we calculated mean probeset hybridization intensities as a function of distance from the 3' and 5' edge of the targeted mRNA molecule. The results are shown in Figure 7. It is evident that the intensity of the signal increases depending on the distance from the polyA site. No such effect is seen for the distance from transcription start site (5'). This effect is further illustrated in Figure 8, which shows that Exon Array gene expression levels are highly correlated with gene length, i.e. short genes appear to be expressed at lower levels than long genes, which is most likely caused by relatively lower efficiency in amplifying short mRNA molecules.

We also noted that the ability of the Exon Array to detect hybridization above background noise levels is not uniform across transcripts. The Exon Array allows the calculation of DABG p-values, which estimate the probability that signal originates from the background noise distribution, rather than true gene expression. In general, probesets with DABG values lower than 0.05 can be accepted to represent true signal. Average DABG values are least significant at both 3' and 5' ends of the gene. The reduction at the 3' end results from the reduced signal intensity levels described above. The reduction at the 5' end is more puzzling, in the absence of a corresponding reduction in signal. We hypothesize that the 5' effect is most likely the result of an elevated GC content of probes located close to promoter regions which are generally unmethylated, GC-rich and enriched in CpG islands [29,30]. In fact the DABG trend at the 5' end inversely mirrors the GC content of the probesets (data not shown).

In effect, probesets that are close to the ends of a gene are likely to exhibit response properties different from the rest of the transcript, and hence produce excess false positive results. Such artefacts are difficult to correct using filtering methods, because the terminal probesets in question are usually detected as expressed above background, but do not respond to expression changes as well as those in the remainder of the gene. In the future, it may be possible to correct for the edge bias by improving the amplification protocol, or computational adjustments. However, at this point interesting Exon Array results in the 3' and 5' ends of genes, particularly those obtained from SI or two-way ANOVA analyses, should be treated with extra caution.

## Discussion

The recognition of alternative splicing and alternative isoform expression as an important component in gene expression analysis has prompted the introduction of isoform sensitive microarray platforms. By targeting individual exons, exon junctions, and annotated isoform variants, such platforms possess the ability to profile not only the expression levels of the entire transcript, but also variations in the types of expressed isoforms. The Affymetrix Exon Array 1.0 ST is one of such commercially available platforms. To date, it has been shown that the Exon Array produces gene expression measurements that are comparable with the previous generation 3' targeted arrays. However, little is known about the in-depth level of similarities and particularly differences among WT and 3' based technologies.

This comparison utilizes the well studied brain and reference samples previously used in the MAQC study to determine sources of variability in profiling gene expression using microarrays. These samples are particularly valuable for the purposes of benchmarking the performance of the Exon Array for two reasons: 1) they allow easy comparison of gene expression level measurements with other platforms that have already been tested, and 2) they allow detection of alternative splicing and isoform difference, since neural tissues are known to be particularly prone to alternative splicing.

Our first conclusions concern the utility of the Exon Array as an expression profiling tool. We note that although the Exon Array results are very consistent with 3' profiling methods, the level of agreement between the Exon Array and 3' targeted platforms (Illumina and Affymetrix U133) is slightly lower than the agreement between the 3' platforms. There are at least two reasons for the decreased concordance.

Firstly, the Exon Array uses a whole transcript, randomly primed amplification protocol, while the two other platforms rely on polyA tail priming. As a result, the two approaches amplify a slightly different RNA pool. This is illustrated very well by the example of several histone genes (known to lack a polyA tail), which the Exon Array indicates are expressed at a much lower level in the brain than in the reference, while the other two platforms indicate a uniform very low level of expression of histone transcripts. As far as we know, differences in expression of histone genes across tissues and treatments have not previously been detected by microarray analysis, and this result is only detectable using the WT approach.

Secondly, many of the outliers in the correlation plot (Figure 3) are due to the presence of real variations in the expression of specific isoforms. This is illustrated using a previously noted example of the *ELAVL1 *gene, which showed discordance across platforms in the original MAQC study, as well as in additional new examples (Additional file 1). The detected expression differences of transcript variants may have important biological significance. For example the longer 3' UTR in the dominant *ELAVL1 *transcript in brain has a different set of putative micro RNA binding sites than the shorter 3' UTR in the reference RNA.

It should also be noted that discordant results will often be obtained because of differences in the annotation provided by microarray manufacturers. We circumvented most of such problems here by re-mapping the probes and selecting only a subset of genes that we were confident were correctly targeted by all three platforms, but researchers should keep in mind that the annotations and gene assignments provided by manufacturers contain numerous errors [31]. In the case of the Exon Array, we found that the most common annotation error resulted from joining together distinct transcripts into single meta-probesets, particularly in the case of transcripts that partially overlap. Thus, we recommend that lists of candidates from individual experiments should be carefully curated.

We also outline how the Exon Array can be used to detect alternative splicing and alternative mRNA processing events. Although our analysis methods are not in themselves novel, and most of them have been briefly described elsewhere [12,21], our goal is to convey to the potential users their intuitive appeal and potential pitfalls. The most challenging step remains the decoupling of whole transcript expression, and individual probeset inclusion. The simplest solution to this problem is to consider only the genes that do not change overall expression levels, but contain probesets that exhibit individual variations. Although this approach produces a highly confident set of alternative events, it can result in a huge reduction of the dataset, particularly in case of comparisons across samples with highly heterogeneous gene expression levels. In the case of MAQC dataset, which has been chosen for the exact reason of it's extreme gene expression variability, imposing the restriction of expression fold change of less than 2 reduces the total number of genes considered by 31% (from 17665 to 12198).

A more inclusive approach is to attempt to correct for gene expression differences that may occur concurrently to splicing differences. We discuss two such approaches: 1) the splicing index, which compares probeset inclusion across samples after normalizing by gene expression levels, and 2) two-way ANOVA, where the interaction term between sample type and probeset can be used to indicate differential inclusion of probesets within transcripts. Both approaches suffer from similar systematic biases; they assume a uniform (linear or log-linear) response of each probeset within a meta-probeset. This assumption is violated in many cases, particularly for probesets that hybridize at very high levels (saturated response) or probesets with hybridization levels close to background (poorly or non-responsive). As a result, in the presence of significant gene expression changes, such analyses predominantly indicate three types of events: dead probesets, saturated probesets, and probesets that may be predominantly skipped (alternative), but not necessarily differentially included across samples. All three types of results constitute false positives, and contribute to the high false positive rates of such analyses.

We also point out two major systematic errors. First, we show that hybridization intensity decreases for probesets close to the 3' mRNA ends, an effect that we believe stems from the random amplification protocol used by the Exon Array. We argue that this is not an annotation artefact, but most likely results from the end of template and reduced random priming potential in the first strand synthesis step amplification. As a result, 3' regions of genes are detected at near background levels, and frequently indicate alternative isoform presence using the SI or ANOVA approaches. A similar problem exists at the 5' end of transcripts, where we hypothesize that the reduction in DABG levels is caused by the elevated GC content of the probesets. These problems are particularly troubling, since many cases of alternative polyadenylation and promoter usage may in fact be associated with changes in transcript expression. This may be due to different promoter strength, or microRNA mediated regulation in 3' UTR (as is likely to be the case in the *ELAVL1 *example shown in Figures 4 and 5). Such real and potentially extremely interesting cases may be difficult to distinguish from differences in probe hybridization potential.

Many of the above systematic errors can be avoided by filtering out potentially troublesome subsets of the data: probesets with extremely low variability (saturated), probeset with low inclusion levels (close to background), and genes with extremely high differences in expression levels across samples. However, such filtering decreases the false positive rates at the cost of reduced genomic coverage.

In our earlier studies, we have also pointed out that in many experimental designs, particularly when samples originate from different genetic backgrounds (e.g. different individuals), the presence of sequence variants within probe target sequences may be a very significant source of errors [8,12]. This effect can be especially prominent in eQTL association studies, where we have shown that it can be responsible for a false positive rate > 80% in alternative splicing analysis [32]. Thus, unless all tested samples are isogenic, we highly recommend additionally "masking" all probes containing known polymorphisms before performing the analysis.

## Conclusion

In summary, the WT profiling provides a wealth of valuable information, which is either not available or misrepresented in traditional 3' gene expression arrays. However, it should be noted that the isoform-level analysis of Exon Arrays is significantly more complicated, suffers from higher false positive rates, and requires more manual intervention than traditional gene expression analysis. We strongly advocate visualization of candidate isoform changes in the context of available genome annotation as a means to both reduce false positive rates and interpret the nature of detected variants.

## Methods

### Exon Array Hybridization

The Universal Human Reference RNA (catalogue no. 740000) and Human Brain Reference RNA (catalogue no. 6050) were obtained from Stratagene and Ambion, respectively. The RNA quality was assessed using RNA 6000 NanoChips with the Agilent 2100 Bioanalyzer (Agilent, Palo Alto, USA). Five technical replicates of each sample were hybridized independently at two test sites: McGill University and Genome Quebec Innovation Centre (Montreal, Quebec, Canada) and Virginia Tech (Blacksburg, Virginia, USA). Biotin-labelled target for the microarray experiment were prepared using 1 μg of total RNA. The RNA was subjected to an rRNA removal procedure with the RiboMinus Human/Mouse Transcriptome Isolation Kit (Invitrogen) and cDNA was synthesized using the GeneChip^® ^WT (Whole Transcript) Sense Target Labelling and Control Reagents kit as described by the manufacturer (Affymetrix). The sense cDNA was then fragmented by UDG (uracil DNA glycosylase) and APE 1 (apurinic/apyrimidic endonuclease 1) and biotin-labelled with TdT (terminal deoxynucleotidyl transferase) using the GeneChip^® ^WT Terminal labelling kit (Affymetrix, Santa Clara, USA). Hybridization was performed using 5 micrograms of biotinylated target, which was incubated with the GeneChip^® ^Human Exon 1.0 ST array (Affymetrix) at 45°C for 16–20 hours. Following hybridization, non-specifically bound material was removed by washing and detection of specifically bound target was performed using the GeneChip^® ^Hybridization, Wash and Stain kit, and the GeneChip^® ^Fluidics Station 450 (Affymetrix). The arrays were scanned using the GeneChip^® ^Scanner 3000 7G (Affymetrix) and raw data was extracted from the scanned images and analyzed with the Affymetrix Power Tools software package (Affymetrix). The microarray data has been deposited in the Gene Expression Omnibus Database (GEO: GSE13072).

### Data Pre-processing and Analysis

The Affymetrix Power Tools software package (Affymetrix) was used to quantile normalize the probe fluorescence intensities and to summarize the probe set (representing exon expression) and meta-probe set (representing gene expression) intensities using a probe logarithmic intensity error model (PLIER, [33]) or robust multichip analysis (RMA, [34]). The above procedures were carried out separately for the two test sites (McGill University and Virginia Tech). The raw data (.cel files) was downloaded from the MAQC website for the Illumina and U133 arrays. In order to keep the number of replicates and test sites consistent across platforms, we only used two of the MAQC test sites (a total of 10 technical replicates of each sample). For the probeset-level analysis and alternative isoform detection, we only used the most confident subset of core probesets from the Exon Array.

### Probeset and Gene Mapping

To determine a subset of genes common to the three platforms, we used the mapping provided by the MAQC study [14] to select 12091 probesets common Illumina and Affymetrix U133 arrays. Subsequently, we used the Exon Array probeset annotation and retained only the genes where the Exon Array meta-probeset coordinates contained both the Illumina and U133 probesets. This procedure resulted in 8391 genes with a high confidence concordant mapping across the three platforms.

## Authors' contributions

AB and DB performed the statistical and computational analysis, and prepared the figures. TK and DG carried out parts of the alternative splicing analysis. RJ and JM conceived of the study and supervised the hybridization of the microarrays. JM wrote the manuscript.

## Supplementary Material

Additional file 1 **Examples of discordance between platforms**. The data is visualized using custom tracks within the UCSC genome browser. We also show the location of U133 and Illumina probes for each gene. The table gives the fold change and significance levels for each platform. A. KISS1R, probable polyadenylation difference. WT profiling indicates that the expression change of the coding sequence of the gene is actually in the opposite direction to the change detected by 3' profiling. B. CRTAC1. A whole transcript change which is only detected by the Exon Array, most likely because the 3' methods target a non-variable UTR region. C. PSD3. Expression change detected by all three platforms, but the Exon Array identifies the nature of the isoform change – annotated alternative promoter usage. D. BCAS1. A putative alternative promoter (not annotated) indicated by the Exon Array. Click here for file

Additional file 2 UCSC browser links illustrating probeset level expression differences (fold-change and p-values) for the top 100 isoforms differentially expressed between the brain and reference samples, obtained from the probeset level analysis. Click here for file

Additional file 3 UCSC browser links illustrating the probeset level expression differences (fold-change and p-values) as well as the normalized (SI) differences for the top 100 isoforms differentially expressed between the brain and reference samples, obtained from the Splicing Index analysis. Click here for file
