# Gene expression and isoform variation analysis using Affymetrix exon arrays

**DOI:** 10.1186/1471-2164-10-121

**Published:** 2009-03-23

**Authors:** Amandine Bemmo, David Benovoy, Tony Kwan, Daniel J Gaffney, Roderick V Jensen, Jacek Majewski

**Affiliations:** 1 Universite de Montreal, Montreal, QC, Canada; 2 Department of Human Genetics, McGill University, Montreal, QC, Canada; 3 McGill University and Genome Quebec Innovation Center, Montreal, QC, Canada; 4 Department of Biological Sciences, virginia Tech, Blacksburg, Virginia, USA

## Abstract

Correction to Bemmo A, Benovoy D, Kwan T, Gaffney DJ, Jensen RV, Majewski J: Gene expression and isoform variation analysis using Affymetrix Exon Arrays. BMC Genomics 2008, 9: 529.

## Correction

After the publication of [1], we were alerted to an error in our manuscript. The x-axis labels for Figure Seven (shown here as Figure 1) were inverted. They should read from left to right: "Distance from the 5' end" and "Distance from the 3' end", respectively. This does not affect our original interpretation of the edge bias affect presented in our original publication in any way. We regret any inconvenience that this inaccuracy might have caused.

**Figure 1:**
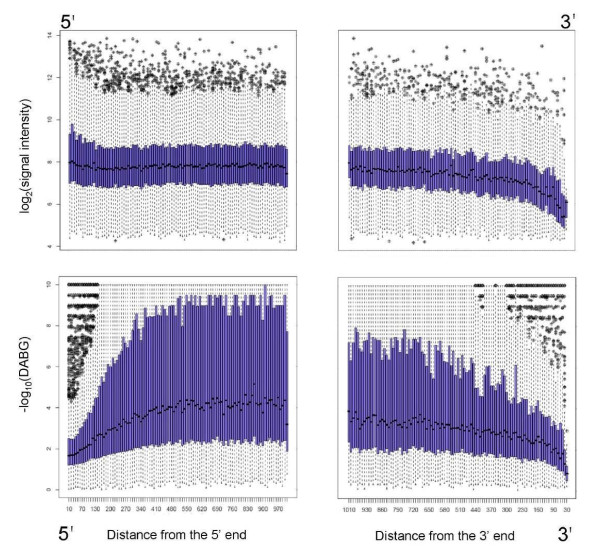
**Edge bias**. This figure illustrates variation of hybridization intensity across transcripts. For each probeset expressed above background levels, we determined the average hybridization intensity as a function of distance from the 5' and 3' ends of the mRNA molecule. Top panels show the average signal intensity as a function of probeset distance from the 5' and 3' ends of transcripts. A slight increase in signal strength occurs at the 5' end while a significant decrease is seen at the 3' end. Bottom panels illustrate the ability of the array to detect the hybridization signal above background levels. Mean DABG values decrease at both 5' and 3' extremities of genes. The 5' effect is most likely the result of increased GC content of the 5' probes located close to unmethylated gene promoters and CpG islands. The 3' effect results directly from the reduction in hybridization intensity. Both effects cause false positive results in Splicing Index and Splicing ANOVA analyses in the presence of changes in expression of the whole transcript. Only genes with detectable expression (average DABG p-value < 0.05) and total mRNA length greater than 1000 nucleotides were included in this analysis. The values were calculated as log-averages of core probeset intensity across all samples. Each point on the plot corresponds to all probeset ending within a bin of length 10 bp, at the indicated distance from mRNA termini.
