# Supplementary figures and images for: Gene Expression and Isoform Variation Analysis using Affymetrix Exon Arrays
Source: BMC Genomics. 2008 Nov 7;9:529. doi: 10.1186/1471-2164-9-529 (PMC2585104; doi:10.1186/1471-2164-9-529)

## Slide 1
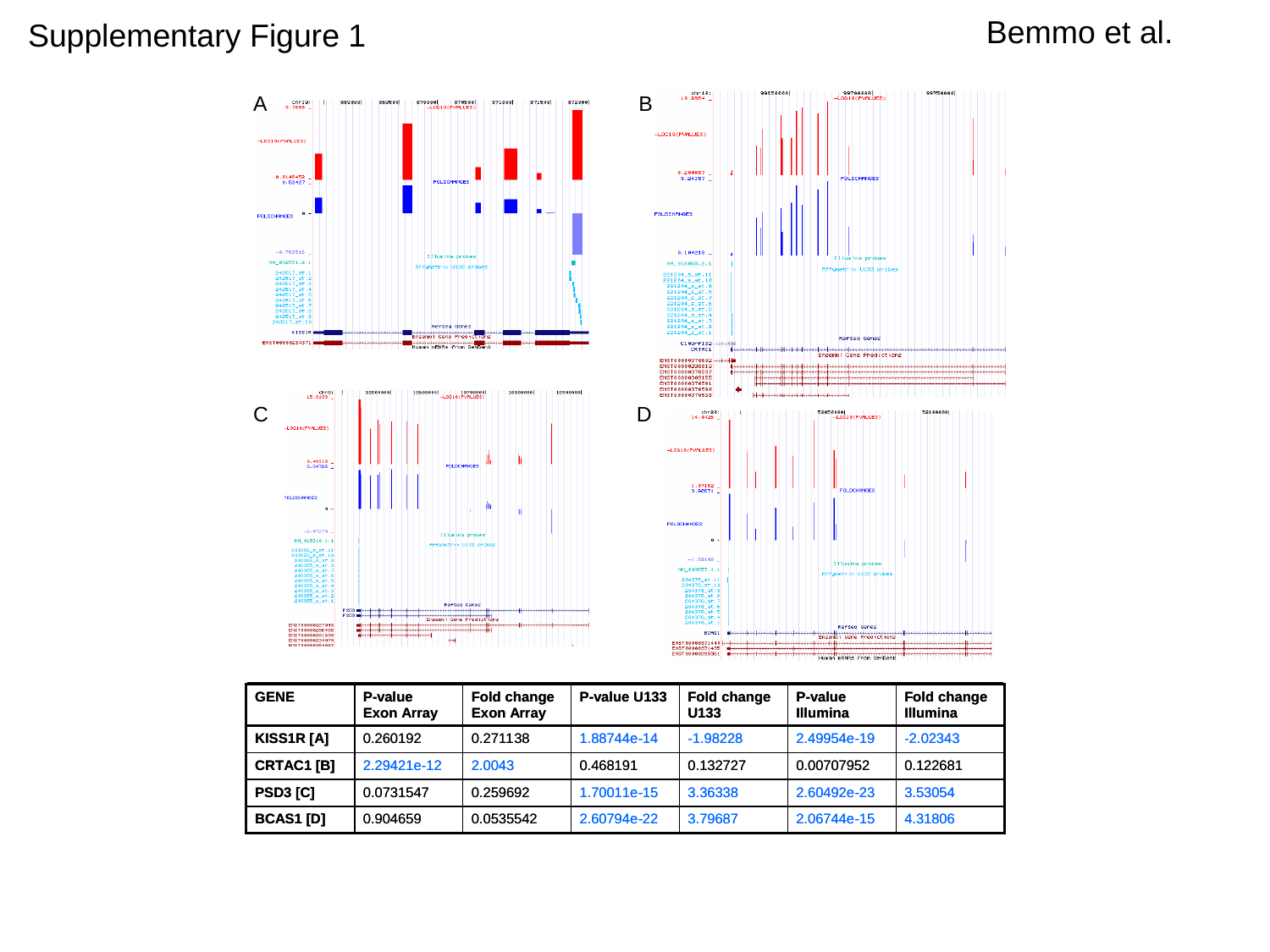

Bemmo et al.
Supplementary Figure 1

Supplement: Additional file 1 — Examples of discordance between platforms. The data is visualized using custom tracks within the UCSC genome browser. We also show the location of U133 and Illumina probes for each gene. The table gives the fold change and significance levels for each platform. A. KISS1R, probable polyadenylation difference. WT profiling indicates that the expression change of the coding sequence of the gene is actually in the opposite direction to the change detected by 3' profiling. B. CRTAC1. A whole transcript change which is only detected by the Exon Array, most likely because the 3' methods target a non-variable UTR region. C. PSD3. Expression change detected by all three platforms, but the Exon Array identifies the nature of the isoform change – annotated alternative promoter usage. D. BCAS1. A putative alternative promoter (not annotated) indicated by the Exon Array. [file 1471-2164-9-529-S1.ppt]
