# Supplementary material for: Gene Expression and Isoform Variation Analysis using Affymetrix Exon Arrays
Source: BMC Genomics. 2008 Nov 7;9:529. doi: 10.1186/1471-2164-9-529 (PMC2585104; doi:10.1186/1471-2164-9-529)
Supplement: Additional file 2 — UCSC browser links illustrating probeset level expression differences (fold-change and p-values) for the top 100 isoforms differentially expressed between the brain and reference samples, obtained from the probeset level analysis. [file 1471-2164-9-529-S2.html]

Link of Genes to UCSC genome browser


| Rank | Name | Id | Accession | Transcript P-value | Transcript Foldchange | Most significant ps\_id | Most significant ps\_pvalue |
| --- | --- | --- | --- | --- | --- | --- | --- |
| 1 | C10orf68 | 3241601 | NM\_024688 | 0.508384 | 0.0474669 | 3241631 | 1.49586e-18 |
| 2 | KRT6B | 3455426 | NM\_005555 | 0.958386 | -0.0265735 | 3455443 | 1.34828e-17 |
| 3 | POU5F1 | 2948863 | NM\_002701 | 0.575534 | -0.110249 | 2948869 | 1.56001e-17 |
| 4 | PTPN4 | 2503109 | NM\_002830 | 0.761105 | -0.051367 | 2503166 | 2.96304e-17 |
| 5 | C10orf97 | 3279410 | NM\_024948 | 0.985292 | 0.00246322 | 3279455 | 8.34807e-17 |
| 6 | ITSN1 | 3918779 | NM\_001001132 | 0.106367 | 0.71544 | 3918911 | 2.96822e-16 |
| 7 | PSD3 | 3126191 | NM\_015310 | 0.0731547 | 0.259692 | 3126205 | 4.86016e-16 |
| 8 | SLC17A3 | 2946106 | NM\_006632 | 0.416292 | 0.0976081 | 2946139 | 1.17673e-15 |
| 9 | CRAMP1L | 3643966 | NM\_020825 | 0.676611 | -0.0806315 | 3644040 | 1.3231e-15 |
| 10 | KIAA0652 | 3329404 | NM\_014741 | 0.14012 | 0.323046 | 3329433 | 2.33568e-15 |
| 11 | HIST1H2AJ | 2947040 | NM\_021066 | 0.264553 | -0.247219 | 2947044 | 3.41336e-15 |
| 12 | BCAS1 | 3910360 | NM\_003657 | 0.904659 | 0.0535542 | 3910363 | 3.60918e-15 |
| 13 | FLJ40504 | 3750578 | NM\_173624 | 0.831525 | -0.167078 | 3750580 | 6.77346e-15 |
| 14 | CACNG8 | 3841157 | NM\_031895 | 0.233166 | 0.490999 | 3841163 | 1.37976e-14 |
| 15 | TPM1 | 3597338 | NM\_001018020 | 0.928129 | -0.026217 | 3597404 | 2.85755e-14 |
| 16 | MTUS1 | 3125915 | NM\_001001925 | 0.0589573 | 0.500099 | 3125960 | 4.58781e-14 |
| 17 | YPEL5 | 2475628 | NM\_016061 | 0.114695 | 1.4014 | 2475643 | 2.98148e-13 |
| 18 | RP11-506K6.1 | 2939232 | NM\_178012 | 0.052901 | 1.02806 | 2939246 | 4.51563e-13 |
| 19 | S100A11 | 2435410 | NM\_005620 | 0.0713496 | -0.143564 | 2435414 | 6.26577e-13 |
| 20 | GH2 | 3766467 | NM\_002059 | 0.23025 | 0.277317 | 3766469 | 7.0098e-13 |
| 21 | NFE2L3 | 2993590 | NM\_004289 | 0.590898 | -0.276842 | 2993612 | 8.87051e-13 |
| 22 | FAM9B | 3998907 | NM\_205849 | 0.457434 | -0.0885693 | 3998929 | 1.08044e-12 |
| 23 | RPS19 | 3834465 | NM\_001022 | 0.639053 | -0.115455 | 3834492 | 1.09877e-12 |
| 24 | DAP3 | 2361036 | NM\_004632 | 0.501971 | 0.228598 | 2361081 | 1.1551e-12 |
| 25 | C18orf17 | 3781980 | NM\_153211 | 0.982898 | -0.00388856 | 3782025 | 1.33057e-12 |
| 26 | NPY | 2993124 | NM\_000905 | 0.09982 | 0.358731 | 2993146 | 1.45231e-12 |
| 27 | LMO1 | 3361672 | NM\_002315 | 0.71166 | -0.0500341 | 3361691 | 1.77254e-12 |
| 28 | KLRC1 | 3444147 | NM\_213657 | 0.915361 | -0.0346912 | 3444150 | 2.4389e-12 |
| 29 | HIST1H4H | 2946383 | NM\_003543 | 0.0790443 | -0.895038 | 2946388 | 2.46118e-12 |
| 30 | CXCL6 | 2731350 | NM\_002993 | 0.737289 | 0.0675584 | 2731355 | 3.86143e-12 |
| 31 | ATP1A4 | 2362950 | NM\_001001734 | 0.0909877 | 0.26739 | 2362958 | 4.41872e-12 |
| 32 | BTBD15 | 3398241 | NM\_014155 | 0.293488 | 0.262013 | 3398280 | 4.59834e-12 |
| 33 | TMC1 | 3174643 | NM\_138691 | 0.174708 | 0.128476 | 3174664 | 4.97387e-12 |
| 34 | PRPH | 3413852 | NM\_006262 | 0.985098 | 0.00364436 | 3413865 | 5.41913e-12 |
| 35 | SHOC2 | 3264004 | NM\_007373 | 0.0507379 | 0.786815 | 3264014 | 7.36639e-12 |
| 36 | PCDHB5 | 2832325 | NM\_015669 | 0.515486 | 0.124178 | 2832331 | 8.74372e-12 |
| 37 | AHCTF1 | 2465324 | NM\_015446 | 0.0586732 | 0.74681 | 2465383 | 8.93101e-12 |
| 38 | RNASE6 | 3527662 | NM\_005615 | 0.247436 | -0.559659 | 3527669 | 9.1493e-12 |
| 39 | ICA1 | 3038065 | NM\_022307 | 0.290942 | 0.313416 | 3038079 | 9.80311e-12 |
| 40 | RPL35A | 2660013 | NM\_000996 | 0.329826 | 0.109109 | 2660019 | 1.13122e-11 |
| 41 | TPTE2 | 3503816 | NM\_130785 | 0.715049 | 0.0884574 | 3503855 | 1.24339e-11 |
| 42 | DPY19L2 | 3459801 | NM\_173812 | 0.29561 | 0.195397 | 3459829 | 1.33335e-11 |
| 43 | PADI3 | 2322818 | NM\_016233 | 0.0721617 | 0.344374 | 2322832 | 1.41125e-11 |
| 44 | PSMA2 | 3047963 | NM\_002787 | 0.760699 | 0.0441803 | 3047983 | 1.43557e-11 |
| 45 | TPD52L2 | 3893760 | NM\_199363 | 0.0534429 | -0.635035 | 3893784 | 1.46654e-11 |
| 46 | FUT8 | 3540552 | NM\_178155 | 0.287761 | 0.211182 | 3540574 | 1.48212e-11 |
| 47 | DPYS | 3147926 | NM\_001385 | 0.0741381 | 0.174506 | 3147957 | 1.58882e-11 |
| 48 | KRTHA6 | 3757037 | NM\_003771 | 0.535264 | -0.356514 | 3757040 | 1.9184e-11 |
| 49 | FLJ10986 | 2338487 | NM\_018291 | 0.762732 | 0.0332099 | 2338507 | 1.92804e-11 |
| 50 | CDK8 | 3482498 | NM\_001260 | 0.186548 | 0.216968 | 3482550 | 2.38337e-11 |
| 51 | PLA1A | 2638077 | NM\_015900 | 0.327186 | -0.140517 | 2638107 | 2.49651e-11 |
| 52 | PFAAP5 | 3508696 | NM\_014887 | 0.795383 | 0.0573404 | 3508704 | 2.79071e-11 |
| 53 | NFAM1 | 3962401 | NM\_145912 | 0.402756 | 0.154735 | 3962407 | 3.69533e-11 |
| 54 | MYLK | 2692447 | NM\_053028 | 0.102728 | -0.222035 | 2692452 | 4.27587e-11 |
| 55 | CYBA | 3704270 | NM\_000101 | 0.0574701 | -1.43001 | 3704285 | 4.78816e-11 |
| 56 | NIPA1 | 3613338 | NM\_144599 | 0.412047 | 0.360973 | 3613340 | 4.86998e-11 |
| 57 | DC2 | 2738949 | NM\_021227 | 0.321677 | 0.366035 | 2738951 | 5.23308e-11 |
| 58 | VPS16 | 3874023 | NM\_080413 | 0.329675 | 0.13592 | 3874105 | 5.28628e-11 |
| 59 | LIMK2 | 3942838 | NM\_016733 | 0.141735 | 0.217562 | 3942879 | 5.89039e-11 |
| 60 | TOMM40 | 3835855 | NM\_006114 | 0.773028 | -0.183545 | 3835864 | 6.45535e-11 |
| 61 | INSRR | 2438612 | NM\_014215 | 0.148732 | 0.187728 | 2438618 | 7.6279e-11 |
| 62 | PC-LKC | 2842624 | NM\_017675 | 0.465165 | -0.119456 | 2842671 | 8.00732e-11 |
| 63 | LGR8 | 3484393 | NM\_130806 | 0.453132 | 0.062186 | 3484404 | 8.94402e-11 |
| 64 | LGALS12 | 3333877 | NM\_033101 | 0.330702 | 0.145717 | 3333881 | 9.10155e-11 |
| 65 | GSG1 | 3445156 | NM\_031289 | 0.827207 | 0.108654 | 3445162 | 9.28802e-11 |
| 66 | MRCL3 | 3776427 | NM\_006471 | 0.616324 | -0.458215 | 3776429 | 9.36363e-11 |
| 67 | COL17A1 | 3305081 | NM\_000494 | 0.501509 | -0.124647 | 3305158 | 1.02661e-10 |
| 68 | MGC10992 | 3693240 | NM\_033212 | 0.483861 | -0.153336 | 3693249 | 1.05165e-10 |
| 69 | PALM2-AKAP2 | 3184408 | NM\_147150 | 0.330425 | -0.222851 | 3184499 | 1.06476e-10 |
| 70 | KCNH6 | 3730698 | NM\_173092 | 0.262879 | 0.0944409 | 3730701 | 1.17412e-10 |
| 71 | C14orf131 | 3553103 | NM\_018335 | 0.177083 | -0.233036 | 3553131 | 1.37597e-10 |
| 72 | CALD1 | 3025545 | NM\_033138 | 0.0761995 | -0.502828 | 3025658 | 1.57045e-10 |
| 73 | B1 | 2996321 | NM\_198428 | 0.077701 | 0.20026 | 2996358 | 1.61953e-10 |
| 74 | EIF5A | 3708422 | NM\_001970 | 0.439167 | -0.155024 | 3708432 | 1.83478e-10 |
| 75 | BSG | 3815014 | NM\_198591 | 0.169152 | 0.394173 | 3815028 | 2.2209e-10 |
| 76 | PPM1A | 3538555 | NM\_021003 | 0.109448 | 0.828373 | 3538569 | 2.2527e-10 |
| 77 | PYHIN1 | 2362351 | NM\_152501 | 0.855256 | 0.0290412 | 2362372 | 2.33454e-10 |
| 78 | CSF3 | 3720675 | NM\_172220 | 0.341682 | -0.155384 | 3720677 | 2.36133e-10 |
| 79 | ATP2B4 | 2375706 | NM\_001001396 | 0.404168 | -0.190466 | 2375764 | 2.40804e-10 |
| 80 | G3BP2 | 2773756 | NM\_203505 | 0.104479 | 0.468925 | 2773765 | 2.49883e-10 |
| 81 | GNPDA2 | 2767790 | NM\_138335 | 0.0970418 | 0.881604 | 2767819 | 2.51146e-10 |
| 82 | DSCR2 | 3932131 | NM\_003720 | 0.789869 | 0.166002 | 3932138 | 2.57883e-10 |
| 83 | HTATIP2 | 3323413 | NM\_006410 | 0.13632 | -0.743994 | 3323430 | 2.87452e-10 |
| 84 | C19orf33 | 3832280 | NM\_033520 | 0.287626 | -0.958408 | 3832286 | 2.98798e-10 |
| 85 | PLEKHF2 | 3107828 | NM\_024613 | 0.0536088 | -0.404892 | 3107833 | 3.04359e-10 |
| 86 | SMAP1 | 2912889 | NM\_021940 | 0.188354 | 0.504211 | 2912921 | 3.52227e-10 |
| 87 | PCGF5 | 3257670 | NM\_032373 | 0.639838 | 0.100923 | 3257700 | 3.60776e-10 |
| 88 | KRTHA5 | 3757020 | NM\_002280 | 0.878312 | 0.015683 | 3757029 | 3.72473e-10 |
| 89 | HLA-G | 2901094 | NM\_002127 | 0.767927 | 0.102948 | 2901102 | 5.14914e-10 |
| 90 | FLJ20433 | 4051619 | NM\_017820 | 0.821301 | -0.060929 | 4051634 | 5.29669e-10 |
| 91 | UBE1 | 3976062 | NM\_153280 | 0.0618094 | -0.255754 | 3976105 | 5.39147e-10 |
| 92 | ZNRF1 | 3668834 | NM\_032268 | 0.223317 | -0.241931 | 3668837 | 5.4734e-10 |
| 93 | GYG | 2647154 | NM\_004130 | 0.635247 | -0.233416 | 2647173 | 5.55004e-10 |
| 94 | USP5 | 3402899 | NM\_003481 | 0.66235 | -0.11071 | 3402925 | 5.98359e-10 |
| 95 | VAMP1 | 3441941 | NM\_199245 | 0.903263 | 0.0451582 | 3441943 | 6.07933e-10 |
| 96 | NUMA1 | 3380901 | NM\_006185 | 0.986656 | -0.00206374 | 3380972 | 6.27117e-10 |
| 97 | OR4X1 | 3330137 | NM\_001004726 | 0.641695 | 0.0723747 | 3330139 | 6.27448e-10 |
| 98 | TNRC6A | 3653398 | NM\_014494 | 0.984322 | 0.00449619 | 3653457 | 6.36079e-10 |
| 99 | ATP6V1C2 | 2469529 | NM\_144583 | 0.868583 | -0.012574 | 2469570 | 6.60968e-10 |
| 100 | H2AFJ | 3406179 | NM\_177925 | 0.178779 | -0.198793 | 3406184 | 6.95474e-10 |
