# Supplementary material for: Gene Expression and Isoform Variation Analysis using Affymetrix Exon Arrays
Source: BMC Genomics. 2008 Nov 7;9:529. doi: 10.1186/1471-2164-9-529 (PMC2585104; doi:10.1186/1471-2164-9-529)
Supplement: Additional file 3 — UCSC browser links illustrating the probeset level expression differences (fold-change and p-values) as well as the normalized (SI) differences for the top 100 isoforms differentially expressed between the brain and reference samples, obtained from the Splicing Index analysis. [file 1471-2164-9-529-S3.html]

Link of Genes to UCSC genome browser


| Rank | Name | Id | Accession | Transcript P-value | Transcript Foldchange | Most significant ps\_id | Most significant ps\_pvalue |
| --- | --- | --- | --- | --- | --- | --- | --- |
| 1 | BAT1 | 2949038 | NM\_080598 | 9.54808e-18 | 4.94499 | 2949051 | 1.63701204072036e-21 |
| 2 | CIT | 3474104 | NM\_007174 | 0.0160324 | 0.518993 | 3474188 | 1.10095921995282e-20 |
| 3 | AQP4 | 3802396 | NM\_001650 | 2.28726e-19 | 6.70218 | 3802414 | 1.38719788156405e-20 |
| 4 | ALB | 2731192 | NM\_000477 | 8.41776e-18 | -7.26839 | 2731229 | 1.61823685835093e-20 |
| 5 | PPP2R2C | 2759205 | NM\_181876 | 9.79339e-18 | 4.75785 | 2759247 | 2.25008610401515e-19 |
| 6 | MICAL2 | 3320717 | NM\_014632 | 8.45377e-09 | 2.00364 | 3320809 | 2.59681643535477e-19 |
| 7 | CAMK2A | 2881300 | NM\_015981 | 4.26709e-21 | 6.05751 | 2881343 | 4.32314625116206e-19 |
| 8 | GPC3 | 4022447 | NM\_004484 | 1.97617e-20 | -5.8824 | 4022584 | 1.48034920419723e-18 |
| 9 | LOXL1 | 3601348 | NM\_005576 | 1.88744e-18 | -3.66363 | 3601356 | 4.31937916725491e-18 |
| 10 | CHN1 | 2587961 | NM\_001822 | 1.86639e-13 | 4.72931 | 2588018 | 4.86212504078515e-18 |
| 11 | TAIP-2 | 2513554 | NM\_024969 | 2.52594e-17 | 4.40157 | 2513607 | 5.98910361787792e-18 |
| 12 | FGA | 2790626 | NM\_000508 | 1.35044e-18 | -5.19682 | 2790628 | 9.27318932072575e-18 |
| 13 | ATP1A2 | 2362892 | NM\_000702 | 1.33714e-15 | 4.74977 | 2362938 | 9.78347952215155e-18 |
| 14 | MAP2 | 2525533 | NM\_031845 | 3.07768e-22 | 5.93517 | 2525585 | 1.1655686206199e-17 |
| 15 | KRT10 | 3756523 | NM\_000421 | 4.98667e-12 | -4.81667 | 3756539 | 1.37392416726567e-17 |
| 16 | IRF3 | 3867980 | NM\_001571 | 9.29781e-07 | -0.616977 | 3867999 | 1.76952147542181e-17 |
| 17 | KIAA0672 | 3710870 | NM\_014859 | 1.75938e-14 | 2.49221 | 3710872 | 2.03327032984006e-17 |
| 18 | C10orf68 | 3241601 | NM\_024688 | 0.508384 | 0.0474669 | 3241630 | 2.43687855135653e-17 |
| 19 | OSBPL1A | 3801621 | NM\_080597 | 0.0207268 | -0.398825 | 3801645 | 3.16416370461785e-17 |
| 20 | CABP1 | 3434490 | NM\_004276 | 2.90834e-19 | 5.14457 | 3434491 | 3.54935921657114e-17 |
| 21 | CHD5 | 2394478 | NM\_015557 | 1.46793e-19 | 2.86197 | 2394557 | 3.84246835052359e-17 |
| 22 | HCAP-G | 2720251 | NM\_022346 | 5.44452e-17 | -4.72009 | 2720287 | 8.85493735538913e-17 |
| 23 | KCNN2 | 2824581 | NM\_021614 | 0.000198385 | 0.834178 | 2824644 | 8.91836424127072e-17 |
| 24 | CACNA1G | 3726618 | NM\_198396 | 1.35089e-14 | 2.57088 | 3726658 | 1.37779610045136e-16 |
| 25 | CNN2 | 3815399 | NM\_004368 | 5.28721e-13 | -3.49152 | 3815415 | 1.67213131459496e-16 |
| 26 | PTN | 3074857 | NM\_002825 | 9.43395e-23 | 4.85541 | 3074902 | 1.7800270331607e-16 |
| 27 | SCN2A2 | 2513471 | NM\_021007 | 6.6996e-15 | 5.59667 | 2513495 | 1.79844748513442e-16 |
| 28 | MAPT | 3723687 | NM\_005910 | 5.16764e-16 | 4.24976 | 3723688 | 1.99376229781273e-16 |
| 29 | TRPM3 | 3209060 | NM\_206947 | 6.30501e-15 | 3.90353 | 3209094 | 2.03027709407547e-16 |
| 30 | PDE4DIP | 2431886 | NM\_001002811 | 6.80713e-15 | 2.58278 | 2431997 | 2.04473766702078e-16 |
| 31 | SLC5A12 | 3366922 | NM\_178498 | 0.00246012 | -0.294375 | 3366937 | 2.05767993340813e-16 |
| 32 | OLFM3 | 2425652 | NM\_058170 | 1.09386e-21 | 4.17871 | 2425671 | 2.44585845717363e-16 |
| 33 | COL6A2 | 3924424 | NM\_058174 | 9.86755e-15 | -3.23425 | 3924479 | 2.48050978095241e-16 |
| 34 | GAD2 | 3239667 | NM\_000818 | 5.43568e-14 | 4.02993 | 3239681 | 2.65867151470433e-16 |
| 35 | DEPDC1 | 2417528 | NM\_017779 | 2.63521e-14 | -3.8482 | 2417535 | 2.7204779206787e-16 |
| 36 | PLEKHB1 | 3339971 | NM\_021200 | 1.50974e-14 | 4.91449 | 3339980 | 3.03627297923878e-16 |
| 37 | ADCY2 | 2800711 | NM\_020546 | 1.49164e-14 | 3.18282 | 2800874 | 3.22686121572251e-16 |
| 38 | DNMT3B | 3882012 | NM\_006892 | 1.96389e-14 | -3.17271 | 3882018 | 3.86843705719742e-16 |
| 39 | PHYHIP | 3127385 | NM\_014759 | 4.55877e-19 | 4.65543 | 3127402 | 4.05004303867111e-16 |
| 40 | AP3B2 | 3636216 | NM\_004644 | 2.60356e-14 | 3.09651 | 3636220 | 4.30983730801687e-16 |
| 41 | DPP6 | 3032647 | NM\_130797 | 1.89252e-17 | 4.09984 | 3032796 | 4.87025733462857e-16 |
| 42 | AURKB | 3744263 | NM\_004217 | 4.47356e-14 | -4.32208 | 3744271 | 5.69436215879188e-16 |
| 43 | GAGE1 | 3977205 | NM\_001468 | 0.000678078 | -0.927299 | 3977210 | 6.55026777616867e-16 |
| 44 | LOC493869 | 2809831 | NM\_001008397 | 1.57225e-14 | -3.26742 | 2809843 | 8.40285747494269e-16 |
| 45 | FAM9B | 3998907 | NM\_205849 | 0.457434 | -0.0885693 | 3998929 | 9.27217744722602e-16 |
| 46 | TYMS | 3775842 | NM\_001071 | 1.97244e-21 | -4.20158 | 3775845 | 9.44065759439082e-16 |
| 47 | PACAP | 2877893 | NM\_016459 | 4.28845e-16 | -3.78232 | 2877900 | 9.53902564813783e-16 |
| 48 | KIAA1409 | 3549264 | NM\_020818 | 5.65767e-15 | 3.68234 | 3549266 | 9.71134708971843e-16 |
| 49 | MADD | 3329724 | NM\_130475 | 1.12812e-05 | 1.35059 | 3329771 | 9.72928936512365e-16 |
| 50 | FAM46C | 2353988 | NM\_017709 | 1.10058e-16 | -3.56141 | 2354006 | 1.03620822690289e-15 |
| 51 | KALRN | 2639552 | NM\_001024660 | 1.98875e-18 | 3.37813 | 2639640 | 1.06337215000486e-15 |
| 52 | SPOCK2 | 3293840 | NM\_014767 | 1.11363e-17 | 4.42269 | 3293880 | 1.09209016680681e-15 |
| 53 | HELB | 3420497 | NM\_033647 | 4.04261e-10 | -1.6124 | 3420529 | 1.09640188214569e-15 |
| 54 | MTUS1 | 3125915 | NM\_001001925 | 0.0589573 | 0.500099 | 3125961 | 1.18107182494882e-15 |
| 55 | PPP4R1 | 3798291 | NM\_005134 | 1.58835e-08 | -1.27268 | 3798331 | 1.30779631830842e-15 |
| 56 | KIF1B | 2319661 | NM\_183416 | 2.0476e-14 | 2.7499 | 2319717 | 1.32679643198564e-15 |
| 57 | NALP1 | 3742783 | NM\_033004 | 2.5631e-08 | -1.52985 | 3742790 | 1.36289549388996e-15 |
| 58 | GTSE1 | 3949055 | NM\_016426 | 7.82728e-16 | -3.39257 | 3949082 | 1.40823547349058e-15 |
| 59 | GPM6A | 2794584 | NM\_005277 | 1.8515e-22 | 7.69209 | 2794651 | 1.40840233898119e-15 |
| 60 | PSD2 | 2831436 | NM\_032289 | 3.39897e-16 | 3.57724 | 2831443 | 1.4956554146361e-15 |
| 61 | TPX2 | 3881443 | NM\_012112 | 7.21847e-15 | -4.42659 | 3881468 | 1.51313700999712e-15 |
| 62 | SRGAP3 | 2662087 | NM\_014850 | 2.05313e-13 | 3.01541 | 2662105 | 1.58510474084411e-15 |
| 63 | SLC12A7 | 2845699 | NM\_006598 | 4.05739e-08 | -1.24337 | 2845740 | 1.63276050852253e-15 |
| 64 | SEPT4 | 3764527 | NM\_080416 | 1.04126e-16 | 5.90008 | 3764556 | 1.72677830693156e-15 |
| 65 | FN1 | 2598261 | NM\_054034 | 1.614e-15 | -4.06231 | 2598262 | 1.81597701726631e-15 |
| 66 | FLJ10719 | 3607510 | NM\_018193 | 5.61077e-21 | -4.22318 | 3607529 | 1.862793375294e-15 |
| 67 | GRM5 | 3385834 | NM\_000842 | 1.43002e-19 | 3.54218 | 3385843 | 1.91822935235119e-15 |
| 68 | KRT6E | 3455516 | NM\_173086 | 1.32686e-06 | -1.35212 | 3455580 | 2.01712531938257e-15 |
| 69 | RIMS1 | 2913123 | NM\_014989 | 1.52044e-11 | 3.7835 | 2913210 | 2.04923506786356e-15 |
| 70 | KIAA0652 | 3329404 | NM\_014741 | 0.14012 | 0.323046 | 3329434 | 2.05399369896124e-15 |
| 71 | IQGAP3 | 2438282 | NM\_178229 | 1.87996e-17 | -2.24152 | 2438285 | 2.22366900961462e-15 |
| 72 | BM88 | 3358393 | NM\_016564 | 1.85134e-16 | 3.59325 | 3358400 | 2.64592806283157e-15 |
| 73 | SPTBN4 | 3833500 | NM\_025213 | 1.40068e-12 | 2.24023 | 3833558 | 2.99141755542355e-15 |
| 74 | MAGI2 | 3058209 | NM\_012301 | 4.87193e-14 | 3.25028 | 3058415 | 3.03737841017451e-15 |
| 75 | CAMK2B | 3048517 | NM\_172079 | 3.37121e-18 | 4.60845 | 3048605 | 3.10859418752257e-15 |
| 76 | PSD3 | 3126191 | NM\_015310 | 0.0731547 | 0.259692 | 3126206 | 3.27005349034582e-15 |
| 77 | PTPN4 | 2503109 | NM\_002830 | 0.761105 | -0.051367 | 2503169 | 3.27671752205834e-15 |
| 78 | NRIP2 | 3440568 | NM\_031474 | 3.12682e-14 | 3.05107 | 3440575 | 3.31130081850355e-15 |
| 79 | TGFBI | 2829947 | NM\_000358 | 2.43292e-17 | -3.76105 | 2829949 | 3.53746814454111e-15 |
| 80 | HCN1 | 2855963 | NM\_021072 | 1.25783e-16 | 4.55045 | 2855966 | 3.98366032544097e-15 |
| 81 | ELAVL3 | 3850960 | NM\_001420 | 1.36841e-14 | 4.37226 | 3850984 | 4.09329391738646e-15 |
| 82 | ATP2B4 | 2375706 | NM\_001001396 | 0.404168 | -0.190466 | 2375766 | 4.21931166763069e-15 |
| 83 | ECE2 | 2655606 | NM\_014693 | 2.62105e-16 | 2.14918 | 2655611 | 4.29076694354459e-15 |
| 84 | BAI3 | 2912416 | NM\_001704 | 5.62186e-20 | 5.46573 | 2912568 | 4.39518620066678e-15 |
| 85 | DGKI | 3074912 | NM\_004717 | 6.71528e-20 | 3.60362 | 3075048 | 4.45598707265365e-15 |
| 86 | KIF23 | 3599811 | NM\_138555 | 4.24392e-17 | -4.14967 | 3599853 | 4.8336828754826e-15 |
| 87 | ANK2 | 2740067 | NM\_001148 | 2.95441e-13 | 4.74717 | 2740217 | 5.38819002978731e-15 |
| 88 | C9orf88 | 3225952 | NM\_022833 | 2.74602e-12 | -2.3821 | 3225987 | 5.55873714910165e-15 |
| 89 | GFAP | 3759410 | NM\_002055 | 3.89163e-24 | 7.29726 | 3759440 | 5.66856937027327e-15 |
| 90 | PPFIA2 | 3463821 | NM\_003625 | 2.17872e-18 | 3.48614 | 3463838 | 5.82057239727337e-15 |
| 91 | DST | 2958325 | NM\_183380 | 1.61e-09 | 1.23406 | 2958467 | 5.95626156970936e-15 |
| 92 | SCN9A | 2585400 | NM\_002977 | 0.000208062 | -0.905439 | 2585446 | 6.12952766478438e-15 |
| 93 | LAMB3 | 2453793 | NM\_000228 | 3.22268e-12 | -1.7825 | 2453816 | 6.24993255120363e-15 |
| 94 | PRPH | 3413852 | NM\_006262 | 0.985098 | 0.00364436 | 3413865 | 6.27778765319056e-15 |
| 95 | GABRA4 | 2768056 | NM\_000809 | 2.06881e-14 | 4.74606 | 2768067 | 6.30124877176361e-15 |
| 96 | DHFRL1 | 2685354 | NM\_176815 | 0.176862 | 0.224464 | 2685364 | 6.91111630543497e-15 |
| 97 | SLC12A6 | 3617312 | NM\_005135 | 0.00285831 | 0.647972 | 3617368 | 6.98627311532837e-15 |
| 98 | LIN28 | 2326485 | NM\_024674 | 1.39808e-16 | -5.8976 | 2326487 | 7.25798476766508e-15 |
| 99 | DTNA | 3784208 | NM\_032979 | 1.45762e-10 | 4.33776 | 3784209 | 7.27686343799348e-15 |
| 100 | MFAP2 | 2398706 | NM\_002403 | 4.8326e-17 | -4.01022 | 2398717 | 7.49494223465947e-15 |
